# Informing Patients With Esophagogastric Cancer About Treatment Outcomes by Using a Web-Based Tool and Training: Development and Evaluation Study

**DOI:** 10.2196/27824

**Published:** 2021-08-27

**Authors:** Loïs F van de Water, Héctor G van den Boorn, Florian Hoxha, Inge Henselmans, Mart M Calff, Mirjam A G Sprangers, Ameen Abu-Hanna, Ellen M A Smets, Hanneke W M van Laarhoven

**Affiliations:** 1 Department of Medical Oncology Amsterdam University Medical Centers University of Amsterdam Amsterdam Netherlands; 2 Department of Medical Psychology Amsterdam University Medical Centers University of Amsterdam Amsterdam Netherlands; 3 Department of Medical Informatics Amsterdam University Medical Centers University of Amsterdam Amsterdam Netherlands

**Keywords:** prediction tool, communication skills training, shared decision-making, risk communication, treatment outcomes, esophageal cancer, gastric cancer, patient-physician communication

## Abstract

**Background:**

Due to the increasing use of shared decision-making, patients with esophagogastric cancer play an increasingly important role in the decision-making process. To be able to make well-informed decisions, patients need to be adequately informed about treatment options and their outcomes, namely survival, side effects or complications, and health-related quality of life. Web-based tools and training programs can aid physicians in this complex task. However, to date, none of these instruments are available for use in informing patients with esophagogastric cancer about treatment outcomes.

**Objective:**

This study aims to develop and evaluate the feasibility of using a web-based prediction tool and supporting communication skills training to improve how physicians inform patients with esophagogastric cancer about treatment outcomes. By improving the provision of treatment outcome information, we aim to stimulate the use of information that is evidence-based, precise, and personalized to patient and tumor characteristics and is communicated in a way that is tailored to individual information needs.

**Methods:**

We designed a web-based, physician-assisted prediction tool—Source—to be used during consultations by using an iterative, user-centered approach. The accompanying communication skills training was developed based on specific learning objectives, literature, and expert opinions. The Source tool was tested in several rounds—a face-to-face focus group with 6 patients and survivors, semistructured interviews with 5 patients, think-aloud sessions with 3 medical oncologists, and interviews with 6 field experts. In a final pilot study, the Source tool and training were tested as a combined intervention by 5 medical oncology fellows and 3 esophagogastric outpatients.

**Results:**

The Source tool contains personalized prediction models and data from meta-analyses regarding survival, treatment side effects and complications, and health-related quality of life. The treatment outcomes were visualized in a patient-friendly manner by using pictographs and bar and line graphs. The communication skills training consisted of blended learning for clinicians comprising e-learning and 2 face-to-face sessions. Adjustments to improve both training and the Source tool were made according to feedback from all testing rounds.

**Conclusions:**

The Source tool and training could play an important role in informing patients with esophagogastric cancer about treatment outcomes in an evidence-based, precise, personalized, and tailored manner. The preliminary evaluation results are promising and provide valuable input for the further development and testing of both elements. However, the remaining uncertainty about treatment outcomes in patients and established habits in doctors, in addition to the varying trust in the prediction models, might influence the effectiveness of the tool and training in daily practice. We are currently conducting a multicenter clinical trial to investigate the impact that the combined tool and training have on the provision of information in the context of treatment decision-making.

## Introduction

### Background

Esophageal and gastric cancers rank eighth and fifth, respectively, in incidence worldwide [1]. The mortality rate is high and, even in the curative setting, the 5-year survival rates do not exceed 50% [2,3]. Over the years, several treatment regimens have come into use, resulting in an array of treatments varying in their effectiveness regarding survival, health-related quality of life (HRQoL), and side effects and complications. For example, localized esophageal cancer can be treated with resection, with or without neoadjuvant chemotherapy or chemoradiotherapy, or with definitive chemoradiation, and localized gastric cancer can be treated with resection with or without adjuvant or neoadjuvant chemotherapy [4-6]. Various options exist for metastasized cancers, with chemotherapy yielding the best survival rates. However, palliative radiotherapy and best supportive care may also be valuable options for specific groups of patients [4,5,7-10].

Oftentimes, the choice between treatment options is based on preferences; the personal weighing of the pros and cons of the options plays a decisive role in the final decision made, and therefore, shared decision-making is needed [11,12]. For shared decision-making to be effective, patients need to be well-informed and thus be offered evidence-based and precise information on treatment outcomes. Evidence-based information refers to the best available, most accurate, and up-to-date evidence. Precise information is concrete, clear, and substantially detailed, such as “In 5 years, 45 out of 100 patients like you that are given this treatment will still be alive.” However, treatment outcomes can differ according to specific patient characteristics (such as age and performance status) and tumor characteristics (such as tumor–node–metastasis [TNM] staging and the number of metastases) [13,14]. Thus, physicians face the challenge of having to inform patients on treatment-related outcomes in a manner that is not only evidence-based and precise but also personalized to the individual patient.

Physicians may face many other challenges when informing patients with cancer on treatment and related outcomes. A vast amount of information on the possible treatment options, including their procedures and associated risks and benefits, must be communicated within the time restrictions of a consultation [15]. Moreover, this information, including schedules, numbers, and probabilities, is often complex and therefore difficult for patients to process [16,17]. Patients’ emotions can complicate information processing even further, especially as esophagogastric cancer is a life-threatening disease [18,19]. Physicians consider dealing with these emotions as a difficult-to-acquire skill [20]. They often worry that their information might even increase a patient’s anxiety or take away a patient’s hope [21-25]. Therefore, physicians may have conflicting opinions and doubts about how to provide precise and numerical information regarding treatment risks and benefits.

Furthermore, tailoring the type and amount of information to the individual patient’s information needs, interests, and concerns (eg, one patient wants to be informed using exact percentages, whereas another would rather get a general description) has also been shown to be a difficult skill for physicians [26]. These challenges impede the ability to meet the information needs of patients with cancer [27-29]. Physicians rarely use clinical outcome data to systematically inform patients, given a certain treatment, on their chances of survival, the most likely side effects, and the consequences on their quality of life [30,31]. However, it has been established that many patients want to receive more information on their treatment-related outcomes and want this information to be more precise [32-36].

Several tools have been developed to aid physicians in this task by using prediction models to generate clarifying visualizations of personalized outcome data, such as the *Predict* and *Adjuvant Online* tools for breast cancer [37,38]. To achieve personalized prediction, these models use multiple characteristics of the patient and the disease to create bar plots and Kaplan-Meier curves displaying survival data. However, to date, no web-based prediction tool exists for use in clinical consultations targeted at patients with esophageal and gastric cancer [39]. Moreover, the probabilities of side effects and HRQoL related to the treatment options are not addressed in the current tools, although patients express information needs related to these outcomes [32,40]. Furthermore, several training programs are available to improve the communication skills of cancer care providers [41,42]. However, these often do not specifically address how to inform patients about treatment options and their particular outcomes, for instance, by using a prediction tool. Combining a prediction tool with communication skills training to address knowledge, attitudes, and skills might increase the usage and adoption of the new tool in clinical practice, improve the overall communication of outcome information by physicians, and stimulate shared decision-making.

### Objectives

Therefore, we aim to develop a web-based prediction tool and supporting communication skills training to improve how physicians inform patients with esophagogastric cancer on treatment outcomes, namely, survival, side effects or complications, and HRQoL. To improve the provision of treatment outcome information, we aim for information that is evidence-based, precise, and personalized to the patient and tumor characteristics, and that is communicated in a way tailored to the individual information needs.

Introducing a change in physician-patient communication by adding a new instrument might initially result in resistance from users, as suggested by behavior change theories [43]. For example, physicians might be reluctant to use the tool because it does not fit into their consultation routine or because they might lack trust in the prediction models. Therefore, our secondary aim is to evaluate the feasibility of the tool and training in practice by consulting physicians, patients, survivors, and experts and to iteratively improve the tool and training.

## Methods

### Overview

Both the tool and training were targeted at physicians in oncology who regularly conduct treatment decision-making consultations. In the development of the tool and training, we focused on patients with metastatic esophageal and gastric cancer. With regard to shared decision-making, this group is confronted with the most complex decision-making process, where personal values and preferences play a large role in deciding among multiple relevant treatment options.

**Figure 1:**
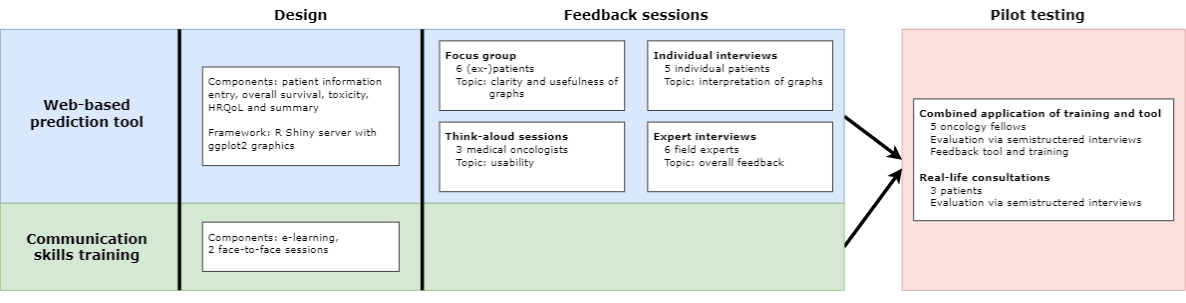
Schematic representation of the development process of the tool and training. HRQoL: health-related quality of life.

The iterative development and testing of this two-part intervention occurred in several phases following the 2008 Medical Research Council framework [44]. This framework provides guidance for developing complex interventions and presents several steps and elements necessary for the successful implementation of the intervention. The framework is divided into the following four phases: (1) development, (2) piloting, (3) evaluation, and (4) implementation. This study describes the first two phases: development and piloting. The development phase is described separately for the tool and training. Both elements of the intervention are joined in the piloting phase as a combined pilot study (see Figure 1 for an overview).

### The Web-Based Prediction Tool: Source

The web-based, physician-assisted prediction tool named *Source*, which contains visualizations of evidence-based, precise, and personalized outcome information, was developed using an iterative, user-centered approach. The tool was designed to be used by oncology health care providers for decision-making consultations. The Source tool, unlike other prediction tools such as *Predict* and *Adjuvant Online*, was not designed for unsupervised use by patients at home to prevent incorrect use, misunderstanding, and lack of emotional support.

First, prediction models were developed to ensure that the Source tool’s treatment outcome information (survival, side effects and complications, and HRQoL) was evidence-based, precise, and personalized to the individual patient and tumor characteristics. Personalized predictions for survival using this tool are based on the SOURCE prediction models [45,46], which predict survival based on the individual patient and tumor characteristics and are regularly updated when new data become available. Depending on the tumor location, either nine variables (for gastric cancer) or 13 variables (for esophageal cancer) are required for predictions. These variables include age, tumor staging, and metastasis characteristics. The model output is the probability of survival up to 2 years following diagnosis and allows for the comparison of multiple treatments.

The side effects (toxicity) of chemotherapy treatment are based on TOXView meta-analyses [7]. These models establish the probability of adverse events such as nausea, alopecia, and neuropathy, stratified by mild or severe grade toxicity (according to the Common Terminology Criteria for Adverse Events [47]), for various chemotherapy regimens. These probabilities are not personalized to the individual characteristics and do not vary over time, as this was not possible with the available data.

Finally, predictions of HRQoL are available from a meta-analysis and describe the change in the EORTC QLQ-C30 (European Organisation for Research and Treatment of Cancer quality of life questionnaire C30) on the global health scale for best supportive care and chemotherapy in metastatic patients up to 6 months after diagnosis [10].

Next, these models and meta-analyses were used to visualize treatment outcomes. For the visualizations to be easy to understand for patients, a previous systematic literature review on visual risk communication was consulted [48]. Furthermore, the literature about usability and usability guidelines for web-based applications [49-52] and existing prediction tools, such as *Predict* and *Adjuvant Online*, were consulted [37,38]. On the basis of the literature, the first set of requirements for the tool was created according to the MoSCoW (Must Have, Should Have, Could Have, Won’t Have) system [53]. This process resulted in the requirements listed in Textbox 1.

**Overview of the requirements of the web interface according to the MoSCoW (Must Have, Should Have, Could Have, Won’t Have) system.**

**Must Have**

1. After opening the tool, a data entry form is shown to enter the variables needed for the prediction models.
2. The data entry form is dynamic and shows only relevant variables.
3. Survival, adverse events, and health-related quality of life outcomes are displayed in their own tabs, and only one outcome is displayed at a time.
4. The outcomes are displayed graphically in a screen-filling image.

**Should Have**

1. The data entry form contains input validation to avoid mistakes during entry.
2. The data entry contains explanations of the variables.
3. The plots can be tailored to the patients’ and physicians’ preferences (eg, time frame and treatments to be compared).

**Could Have**

1. The tool’s display language can be set to Dutch or English.
2. A textual summary accompanying the plots can be generated and printed so the patient can review the information at a later time.
3. A help function for physicians is incorporated.

A prototype of the web interface was created based on the literature guidelines and first requirements. The web-based tool was developed using the RShiny software (version 1.2.0; RStudio) supplemented with ggplot2 (version 3.2.1) to create graphs [54,55]. The creation, evaluation, and improvement of the tool followed an iterative user-centered design framework, where feedback was gathered from end users (patients and physicians) and experts. By iteratively updating the tool, we aim to provide improvements for the tool after each feedback session and avoid receiving the same feedback after each feedback round. A total of 4 feedback sessions were conducted from January 2018 to July 2018.

First, a face-to-face focus group was conducted with 6 patients with esophageal and gastric cancer and survivors from the Foundation for Patients with Cancer in the Digestive system after verbal informed consent was provided. The aim of this focus group was to obtain feedback on the tool in a group setting and promote discussions among the group members. One of the researchers acted as a moderator and presented the participants with each of the tool’s graphs. Each displayed graph was accompanied by a short oral explanation, after which the participants were asked for their opinions. Feedback on the web interface and suggestions for improvement supported by multiple participants were used to create an improved version of the tool. The focus group session was audio-recorded and analyzed according to microinterlocutor analysis to systematically evaluate the participants’ remarks [56].

In a second feedback round, 30-minute, semistructured, face-to-face interviews were conducted with individual patients with esophageal and gastric cancer. The main focus of these audio-recorded interviews was to determine whether the patient interpretation of the revised graphs was adequate. By conducting interviews with individual patients rather than in a group, the aim was to review patient interpretations without the influence of other patients. The interviews were conducted using a piloted script. A total of 5 outpatient participants were recruited by an oncologist at the Amsterdam University Medical Centers. Following the participants’ informed consent, 2 researchers presented the tool to patients by using fictitious predictions of treatment outcomes. Patients were asked to interpret the presented graphs and describe their meaning to assess their understanding. Thereafter, the researcher provided the correct description of the graph, and the patients’ subsequent feedback was gathered. Feedback was registered in a response matrix, including the frequency of different remarks, to establish which possible improvements could be implemented.

The third feedback round aimed to evaluate the usability of the tool when used by medical oncologists, and 3 medical oncologists at the Amsterdam University Medical Centers participated in individual face-to-face think-aloud sessions. After providing informed consent, they were asked to use the tool for two paper patient cases while stating out loud whatever came to mind. The cases described fictitious patients with esophageal or gastric cancer, including some of their clinical characteristics. Several tasks and questions about specific outcomes (eg, “What is the 1-year survival probability with best supportive care?” and “Which treatment has the best quality of life after 6 months?”) were posed to guide the use of the tool by medical oncologists. At the beginning of the think-aloud session, a video explaining the think-aloud method [57] was presented and the participants were asked to complete a short practice exercise to ensure that they understood the think-aloud method before starting the task. After the think-aloud session, participants completed the System Usability Scale (SUS) to measure the ease of use and overall likeability of the web-based tool [52]. Both screen captures and audio recordings were registered during the think-aloud session. One of the researchers (FH) used both recordings to register whether the oncologists successfully completed the tasks, how many mouse clicks they used to complete a task, and which buttons they clicked on the web interface. The median SUS scores were calculated to provide a quantitative indication of usability.

In the fourth and last round, feedback from experts was gathered by conducting semistructured interviews with 6 researchers with expertise in patient-physician interaction, shared decision-making, risk communication, medical informatics, and clinical decision support software. The experts were presented with a walkthrough of the tool and its options. Interviews were recorded and summarized to determine which possible improvements were brought forward.

### The Source Supportive Communication Skills Training

#### Overview

Communication skills training was developed to educate physicians on informing patients with cancer in a treatment decision-making consultation using the Source tool. Due to the complexity of the skills needed, it was important to specify clear learning goals. As stated in complex learning theory, when training complex skills, the desired learning outcomes must address the following domains: knowledge, attitudes, and skills [58-60]. The training aimed for physicians to be able to name the most important tips and tricks for adequately informing patients on treatment outcomes and communicating treatment risks and benefits (knowledge). Furthermore, the training aimed for physicians to have a positive outlook on using numbers to inform patients about treatment outcomes and their ability to inform patients in an evidence-based, precise, personalized, and tailored manner (attitude). Moreover, the training aimed for physicians to be able to use the Source tool and to incorporate the tool to inform patients during consultations (skills). Finally, the training aimed to increase physicians’ ability to provide information tailored to patients’ informational needs and level of understanding (skills). A team (n=5) of experts in medical communication and psycho-oncology and experienced trainers in medical communication discussed the context and content of the training and set learning objectives. In addition, the literature on training and shared decision-making frameworks was reviewed.

As physicians value time-efficient and flexible training [16], the training was designed as blended learning, encompassing preparatory e-learning and a face-to-face component. The 4-step shared decision-making model proposed by Stiggelbout et al [12] was used as a framework. This model distinguishes the following four essential steps for shared decision-making: (1) setting the agenda, (2) informing about treatment options, (3) exploring patients’ values, and (4) making a decision in agreement [61]. The outline of the training was based on previous communication skills training for skills in shared decision-making, as designed for and proven to be effective in the CHOICE (Choosing Treatment Together in Cancer at the End of Life) trial [61] and the literature on the guidelines for effective communication skills training [42,62]. The focus of the Source training is the second step of this model, that is, informing patients about treatment options and the pros and cons thereof.

#### e-Learning

First, e-learning was targeted at summarizing the evidence base for effective information provision and providing physicians with tips and tricks for clinical practice. to this end, we consulted the literature related to theories, evidence, and guidelines on the provision of information in medical practice [12,63-67]. The assembled literature and theories were summarized into short chapters, each covering a different subtopic. The expert team discussed the scripts in these chapters to obtain a consensus on the frameworks and models used. Interactive elements, such as exercises, were added to the e-learning to enable the learner to actively process the information. Second, the e-learning aimed to introduce the Source tool, thereby addressing the use and functionalities of the tool and the underlying prediction models.

An earlier study concluded that physicians value both visual attractiveness and variation between learning activities in e-learning [16]; therefore, the layout, animations, and videos were developed in cooperation with a small visual design company, Public Cinema.

#### Face-to-Face Sessions

Face-to-face sessions were developed based on previous experience in developing and evaluating communication skills training in oncology [42,61,68]. The most important recommendations from these earlier studies were to role-play with an actor to practice the lessons learned during the training and provide the trainee with personal feedback [42]. Development took place in multiple sessions with the expert team. The basic assumptions for effective information provision, as incorporated in the e-learning, served as a starting point for the training content. Derived ideas were written down and discussed to create a training script and a supportive PowerPoint presentation. The casuistry for the training actors was developed together with a clinical expert (HWMVL). These multiple development sessions led to a conceptual version of the training.

### Pilot Study Tool and Training

A pilot study was conducted from December 2018 to March 2019 to test both the tool and training in a real-life setting. As this pilot study targeted patients with advanced disease only, we included medical oncologists and metastatic cancer patients as study participants. The pilot study was evaluated by the Medical Ethics Review Committee of the Academic Medical Center Amsterdam (reference number: W18_278). In total, 5 medical oncology fellows (2 men and 3 women) from two university medical centers were invited to use the tool and trained according to the concept training format. After completion of the training, participating fellows were individually interviewed via telephone in a semistructured manner to gather feedback to improve both the Source tool and training. For the tool, the focus of the feedback was on opinions and experiences regarding usability and willingness to use the tool. Regarding training, feedbacks on the different components, training as a whole, and perceived utility were collected.

In addition, an experienced medical oncologist (HWMVL) conducted three treatment decision-making consultations with outpatients using the Source tool for information provision and in line with the training principles. These consultations were recorded on video after obtaining written informed consent from the patients and oncologists. To comply with ethical standards and according to the training, only information that the patient wanted to receive was disclosed to the patient. One-on-one semistructured interviews were conducted with the 3 patients by one of the researchers (LFVDW) to gather their experiences with the physician’s outcome information and the use of the Source tool.

## Results

### The Web-Based Prediction Tool: Source

A prototype web interface was created based on the findings of a systematic review of the effects of different types of risk communication on patients with cancer [48]. Following this review, we decided to use clear and precise risk information (eg, percentages or frequencies) and simple graphs with a limited amount of information displayed. As the review did not yield consistent guidelines on which types of graphs to use, it was decided to visualize the outcomes in multiple ways. In this way, graphs can be used according to the preferences of individual patients, and the amount and presentation format of the information displayed can be tailored to their needs and preferences. The resulting RShiny web interface runs on an x64 Linux server (version 3.10.0).

### Source Tool Components

**Figure 2:**
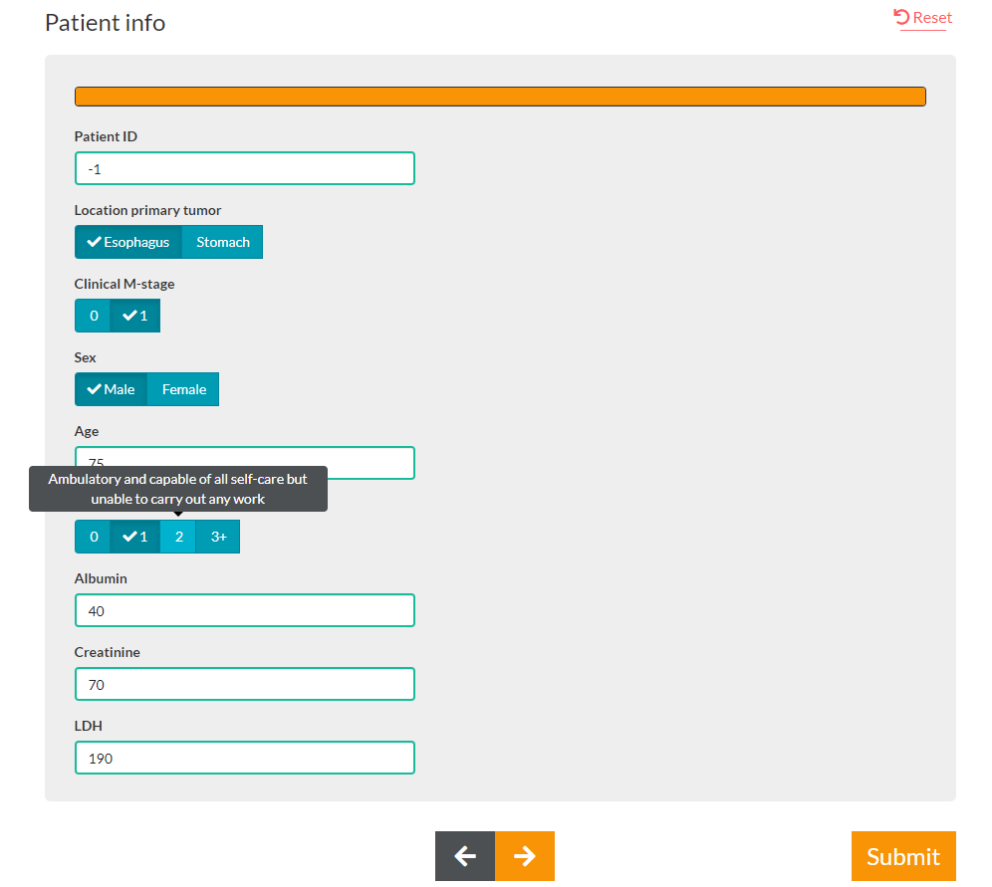
A screenshot of patient data entry. The data entry screen displays the fields that are necessary for the prediction models and meta-analyses. Additional information on variables such as the World Health Organization performance status is provided with a mouse-over.

The final tool, Source 1.103, contains five main components. The first component, the patient information entry component, allows the oncologist to enter the patient characteristics necessary for the prediction models and meta-analyses, using supporting information, such as the definitions of TNM variables (Figure 2).

**Figure 3:**
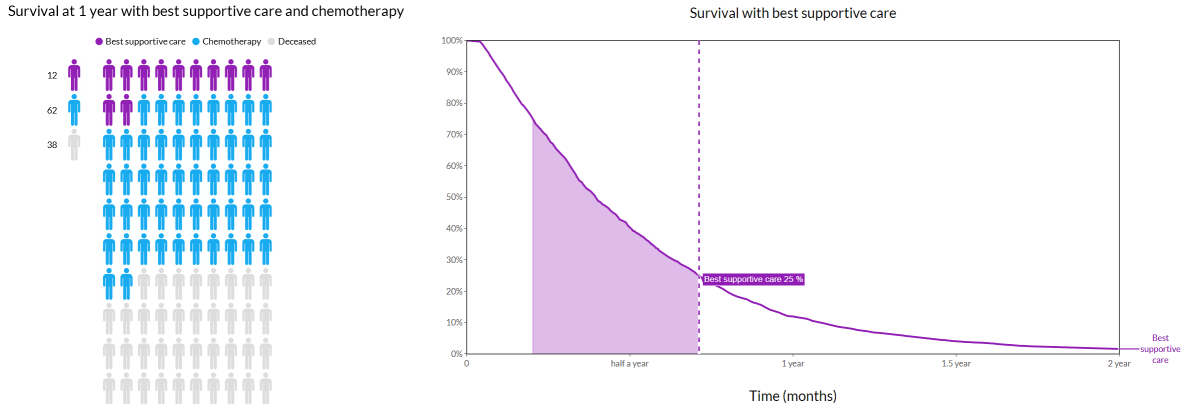
A screenshot of survival graphs. On the left, a pictograph displaying the predicted survival for best supportive care and chemotherapy after 1 year is shown. On the right, the Kaplan-Meier curve for the best supportive care is shown. The optional shaded area displays the so-called typical outcome scenario (with survival ranging from 25% to 75%).

The survival component outcome was visualized in two ways: an icon array displaying the survival probability at a given point in time by coloring a subset of 100 figures and a Kaplan-Meier curve (line graph) displaying the survival probability over time (Figure 3). The survival component incorporates the possibility of switching between the two presentation formats. From the options menu, it is possible to select specific treatments for comparison (eg, best supportive care and chemotherapy) and change the time frame of the prediction (from 6 to 24 months) to show a per treatment CI and three survival scenarios (indicating the best-case scenario comprising the top 25%, the worst-case scenario comprising the bottom 25%, and the typical outcome comprising the middle 50% [63,64]).

**Figure 4:**
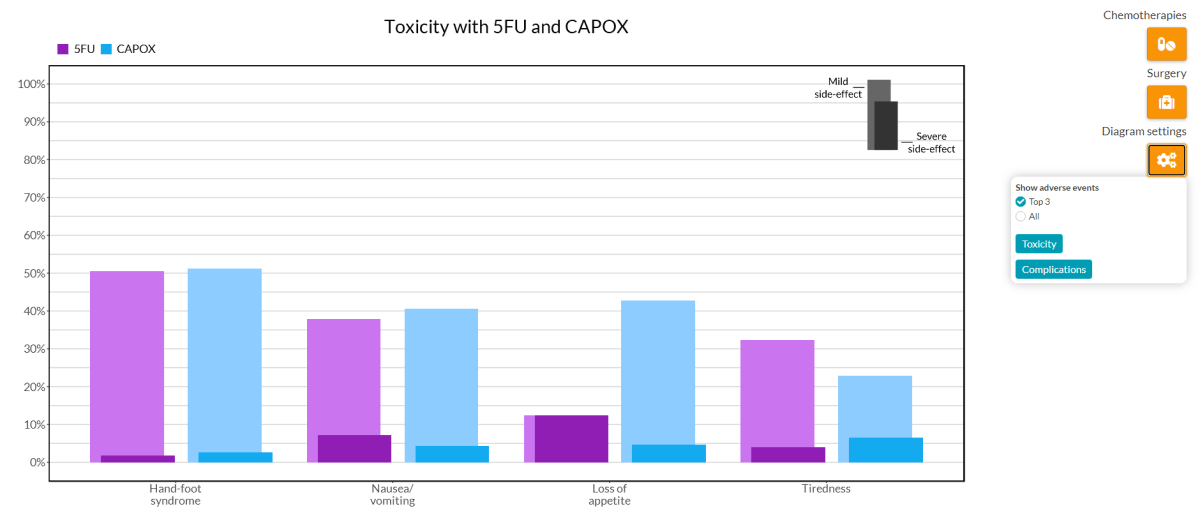
A screenshot of the side effects bar chart. This displays the three most commonly occurring toxicities for both 5FU and CAPOX. The darker bars indicate severe toxicities, and the lighter bars indicate mild toxicities. 5FU: 5-Fluorouracil; CAPOX: capecitabine combined with oxaliplatin.

The side effects component displays bar charts for various toxicities (Figure 4), as the meta-analysis provided static probabilities for each of the adverse events [7]. Each side effect was visualized by two stacked bars, one for mild side effects and one for severe side effects on the Common Terminology Criteria for Adverse Events scale [69]. The side effects of multiple chemotherapy regimens can also be compared. To avoid information overload, only the three most frequently occurring side effects are shown initially, although it is possible to select all side effects.

**Figure 5:**
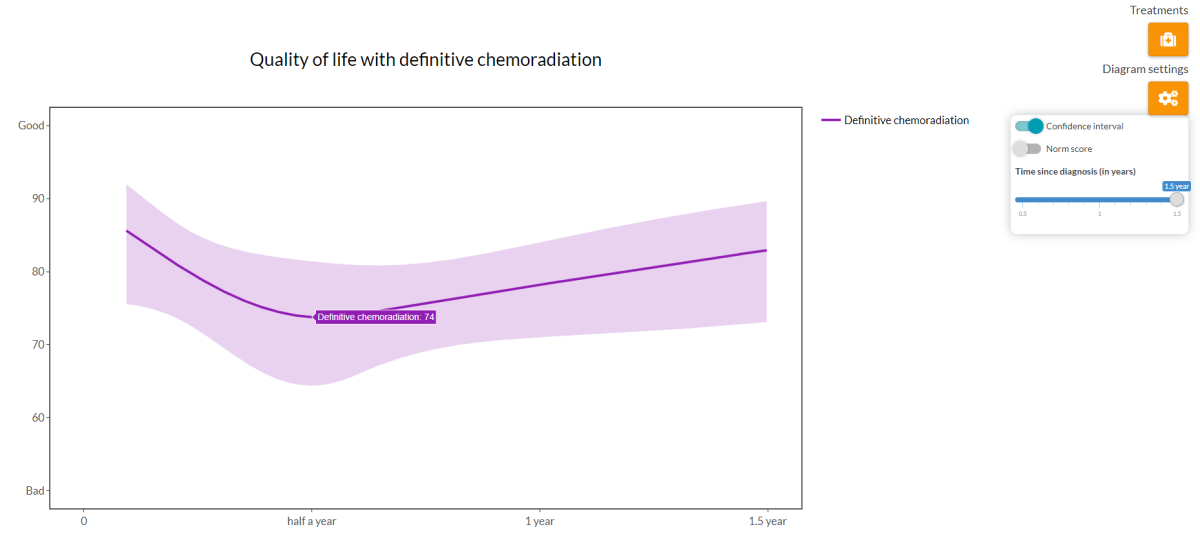
A screenshot of the HRQoL (health-related quality of life) line graph. The graph displays the HRQoL following definitive chemoradiation. The shaded area optionally displays the CI of the HRQoL line.

HRQoL is displayed in a line graph and shows the EORTC QLQ-C30 global health score over time (Figure 5). There are options to compare HRQoL in best supportive care with chemotherapy and display a CI and reference value (obtained from the European Organisation for Research and Treatment of Cancer reference values manual [70]). The final component is a summary that can be printed as a handout for the patient or saved as a PDF file. This feature enables physicians to show the aforementioned graphs accompanied by an explanatory text. This text is dynamically generated using the selected treatment data and explains the content of the graphs.

### Evaluation Round Feedback

The four evaluation rounds of the tool resulted in several minor visual and functional adjustments to the web interface, as described in Multimedia Appendix 1. Major adjustments to the tool resulting from the gathered feedback were mostly adjustments regarding usability, such as increasing the font size and positioning of the legend. For the survival outcome, the icon array was found to be the most comprehensible, whereas the line graph provided the most insight for survival over time. Therefore, it was decided to keep both formats for the survival outcome, as the graphs supplemented each other. The line graph also remained in the tool as it incorporated the scenario’s functionality (indicating worst-case, typical, and best-case survival), a feature that was found important by most patients. For side effects, it was found that the patients did not correctly interpret the meaning of the stacked bar charts. The bars were changed into two nonstacked bars with 90% overlap to display mild and severe adverse events for the same side effects to increase clarity on the meaning of the graph (Figure 4). Furthermore, it was decided to remove bar charts as a display option for HRQoL data, as both patients and oncologists found the graph unclear and wanted the data to be displayed over time, as HRQoL may increase and decrease over time. Showing the predictions at a single time point may therefore not provide sufficient insight. In the final design, various options are available to personalize the displayed graphs. Regarding the usability of the tool, it received a median SUS score of 90.0 out of 3 ratings (above the *Excellent* threshold of 80.3 points [52]) during the think-aloud sessions.

In the pilot study, 4 of the 5 oncology fellows participated in an evaluative semistructured interview following the training. The fifth fellow did not respond to repeated invitations. All 4 oncology fellows reported that the tool was highly usable overall. Some minor suggestions were provided for improving the display of certain options, graphs, and buttons. Of the 4 oncologists, 3 reported that they would use the tool in their clinical practice. They especially valued the personalized nature of the tool’s predictions and the clear and easy-to-understand visualizations for patients. Furthermore, the inclusion of HRQoL data and the option to print a summary for the patient were found useful. The fourth fellow would like the prediction models to be further developed before using the tool. For instance, during the pilot training and interviews, several critical remarks on the prediction models were expressed, such as the lack of World Health Organization performance status as a predictor of overall survival. These comments likely reflect a possible lack of trust in the underlying models and analyses of the tool. Another barrier to using the tool that one of the fellows addressed was the fear of emotionally confronting the patient with the exact numbers. This fellow did indicate that this fear was overcome through the tailoring skills that he had acquired during the training.

### The Source Supportive Communication Skills Training

#### The e-Learning Module

A short and to-the-point e-learning module was developed to provide an overview of the theoretical background on the provision of information in a treatment decision context and introduce a web-based prediction tool. The e-learning module starts with a short peer endorsement video of the training of physicians that discusses the Source tool. Subsequently, physicians can navigate through 4 chapters. The first chapter provides an overview of the principles of effective information provision in the context of a shared decision. The second chapter introduces the physicians to the Source tool by presenting them with a tailor-made instructional video of its use and functions. Furthermore, a summary of the tool’s models and their underlying data is provided. The third chapter provides an overview of tips and tricks for informing patients about the risks and benefits of treatment (Multimedia Appendix 2). The final chapter consists of a short and practical summary of key take-home messages. In all chapters, textual information and short assignments are alternated by instructional videos and animated knowledge clips. A simple and appealing visual design is applied.

#### Face-to-Face Training Sessions

The face-to-face component of the training consisted of two group sessions of 3.5 hours each, provided by an experienced trainer, with approximately 2 to 3 weeks in between to facilitate intermediate practice. The sessions were aimed at small groups of 2 to 6 participants, as this approach enables every participant to practice and receive personal feedback. Such a setting can also promote interactivity [71]. Both sessions involved individual role-play exercises with a professional actor in which feedback was provided by the trainer, the actor, and peers to learn additional skills [62]. Furthermore, group discussions were stimulated and led by the trainers. This approach was used to encourage physicians to discuss their attitudes toward using numbers and the tool in the context of the provision of information to patients [62,72].

The first session covered the skills of setting the agenda of the decision-making consultation, introducing the tool and informing patients on survival outcomes. Physicians were asked to practice separate parts of the consultation while receiving feedback from other physicians and the trainer. Physicians were instructed to use the acquired knowledge and skills during their outpatient consultations before the next training session. The second session allowed for the repetition of issues addressed during the first session and sharing experiences of applying the lessons learned of the first session in clinical practice. Next, skills were addressed, again with role-play and feedback, to inform patients about treatment outcomes in terms of side effects and complications and HRQoL. This session also addressed how to conclude decision-making consultations.

In both sessions, tailoring of the amount and type of outcome information to specific patients played a significant role in both role-play practice and group discussions. Tips and tricks were discussed regarding how to determine an individual’s information needs and wants, how to fit these needs with the informational needs of the physician, and whether and how the tool could contribute to tailored information giving.

The total duration of the blended learning was 7.5 study hours, which consisted of 0.5 hours of e-learning and 7 hours of face-to-face training.

### Feedback and Major Adjustments

#### Medical Oncology Fellows

Fellows reported that they enjoyed participating in the training and specifically valued personal coaching and practical tips. Furthermore, fellows appreciated the trainers and actors. In their opinion, there was a good balance between the information provided by trainers and practical exercises. Fellows especially appreciated the feedback during the training from the actor and trainer on their role-play with the actor. Overall, the training was described as useful, and specific improvements were suggested.

A point of improvement that was brought up was the substantial time investment in the training. In particular, the pace of the e-learning and instructional video was considered too slow. Furthermore, the timing of the face-to-face sessions following a day of work was considered inconvenient. The most important adjustments to the training as a result of the fellows’ feedback were related to accelerating the pace and adding an individual booster session to the training in which the physician could receive personal feedback from one of the trainers on a full, recorded consultation.

#### Patients With Metastatic Cancer

Despite their emotions on the subject, 2 out of the 3 patients were willing to participate in a short interview about their experiences with the consultation. Both appreciated the use of the Source tool and the physicians’ explanations of possible treatment outcomes. Patients expressed differences in their experiences regarding the amount of information about treatment options and outcomes. Although one patient reported being satisfied with the amount of information, the other indicated that the amount of information was too extensive for him to memorize it all. He needed a printed summary of the tool for support. Both patients mentioned their struggle with the meaning of risk or benefit for themselves, as great uncertainty remains about their own future, despite the information provided.

## Discussion

### Findings

Our study shows the iterative development and pilot testing of the Source tool and training. This combined intervention was developed using scientific evidence and input from physicians, patients, and experts. This process resulted in the first web-based prediction tool to inform patients with esophagogastric cancer during consultations on survival, side effects, and HRQoL of different treatment options. Furthermore, we created a supporting training to teach physicians the communication skills needed to use the tool and to provide patients with information in an evidence-based, precise, personalized, and tailored manner. Preliminary evaluation results are promising and provide valuable input for further development and testing of both elements.

Both the tool and training were valued by participating physicians and patients. Physicians especially appreciated the practical approach of the training; the multiple practice opportunities and personal feedback helped them use the tool. Nevertheless, despite their positive attitudes toward the tool and training, old habits could stand in the way of using the tool and may impede the use of learned communication techniques in clinical practice. Behavioral change theories show that many factors can contribute to but also stand in the way of learning new behaviors. Resistance could, for instance, arise as a result of a different expected outcome of the tool or because of a low tolerance for change [43]. The *transfer* of training describes the possible behavioral change resulting of an educational intervention such as training. From the literature, we know that although certain trainee characteristics (such as the perceived utility of the training) and training design factors (such as a realistic training environment) can promote the transfer of training, they are also strongly influenced by characteristics of the work environment, such as situational cues (eg, social support from peers or supervisors) or consequences (eg, negative or strong emotional reactions from patients) [73]. These characteristics can be difficult to control in the setting of everyday hospital care. However, concerning the training design factors, the distribution of the training sessions might be an important factor contributing to the transfer of this tool and training for daily clinical practice. Indeed, the so-called *spacing* between the two face-to-face sessions and the booster session of the training might help increase task performance [74-79].

During the pilot training, it was noted that in some cases, the fellows lacked trust in the prediction models used in the tool. Further steps were taken to increase the physicians’ trust. For instance, details about the underlying data and publications were added to the tool to provide more information about the methodology and sources. Model updates (such as the 2020 version of the survival model), which increases the model’s performance and sample size and includes, for instance, World Health Organization performance status as a predictor, may also increase trust in the tool [39]. Finally, external validation of the models can also generate trust in the validity and applicability of the tool [80].

The use of the Source tool could also be influenced by the application’s usability and how well the tool solves the patient-informing problem as perceived by patients and physicians. Therefore, iterative usability testing is necessary to achieve an acceptable level of usability. As the number of testing rounds in this study is limited compared with other studies [37,38], the tool’s usability may be further improved. This issue will be an ongoing point of attention that we will address during future testing and development of the tool. From a patient’s perspective, some uncertainties regarding treatment outcomes may be reduced during the consultation, whereas other uncertainties remain. For example, the tool might support patients in participating in shared decision-making, but active participation in this difficult choice might also overwhelm them [81]. These issues can be addressed in medical education and training by dealing with a broad spectrum of patient uncertainty.

On the basis of our experiences, we can provide several recommendations to aid future research in creating and evaluating web-based prediction tools with training. First, we advise involving end users, such as patients and physicians, in the early stages of development. Assumptions and implementations are often made from the perspective of developers, which may not coincide with the needs and wishes of patients or physicians. By evaluating at an early stage, it is possible to adjust the tool and training, and subsequent improvements can be implemented more seamlessly. Although not formally evaluated in this study, user research to investigate patients’ and physicians’ ideas and expectations regarding such a tool could also contribute to the usability and adoption of the tool in clinical practice. Second, evaluation by physicians may be complicated because of their busy schedules. We recommend making the feedback rounds with physicians as short as possible, planning them sufficiently in advance, and having them take place on education days. Third, as insights on data visualization and risk communication may change constantly, we recommend facilitating ongoing updates of a designed prediction tool. We also suggest that future research should use the current state of the art when designing a new tool or training. Fourth, we advise that communication on outcomes with subjective interpretations, such as HRQoL, deserves a more prominent place in communication skills training. We noticed in our training that physicians often had trouble explaining outcomes such as HRQoL. Finally, it was observed that end users sometimes had conflicting opinions regarding improvements to the tool or training. As it is not possible to cater to everyone’s wishes, we recommend weighing the pros and cons of suggestions and deciding whether a personalization option will be implemented (such as displaying survival as a pictograph and a line graph) or whether a single option will be implemented (such as the background color of the web interface).

Most of the patients and physicians who participated in this study agreed that the tool and training added value to clinical practice. However, bias may have played a role in the evaluation of the tool and training, as the evaluation only partly took place in clinical practice. To investigate this potential bias and the extent to which the combined tool and training aid in information provision in the context of treatment decision-making, we are currently performing the third phase of the Medical Research Council framework: evaluation, a multicenter effect study (registered under NCT04232735, the SOURCE trial). In this stepped-wedge trial, physicians receive training and use the Source tool both in simulated patient assessments and with outpatients. The effect that the intervention has on the outcome information provided by oncology physicians was quantitatively investigated by recording these consultations before and after the intervention and analyzing physicians’ outcome-related remarks. The primary outcome of this study is the provision of precise outcome information; secondary outcomes include the amount of tailoring to the information needs of patients, the patients’ own knowledge and opinions on the communicated outcome information, and the influence that the consultation has on patients’ emotions. As trust in the SOURCE models was found to be a potential barrier to using the tool in the pilot study, physicians’ trust in the models will be closely monitored and specifically addressed during the trial. The models included in the Source tool are being continuously improved and updated, in part, to address these issues.

In this trial, both palliative and curative patients will participate, and models aimed at potentially curable patients (cM0) will be added to the Source tool. The survival models are based on the 2020 version of the models, which include updated palliative prediction models and newly developed curative prediction models for both esophageal and gastric cancer [39]. The HRQoL model for curatively treated patients originates from a systematic review and meta-analysis, and treatment side effects for curatively treated patients were provided by the COMplot study [82,83]. The addition of these models to the Source tool enables evidence-based and precise information personalized to the individual’s characteristics in the full spectrum of patients with esophagogastric cancer. As the tool is currently being tested in a trial, access is currently restricted to trial participants. However, after the conclusion of the trial, the tool will be freely available in both Dutch and English, enabling the use of the Source tool in clinical practice [84].

### Conclusions

We developed and evaluated a web-based tool and training to inform patients with esophageal or gastric cancer regarding treatment outcomes. Through evaluation and a pilot study, patients and physicians indicated the added value of the tool and training, and both were improved based on their feedback. The tool and training are currently being evaluated in a multicenter trial to determine their added value in clinical practice.

## Appendix

Multimedia Appendix 1 Detailed adaptations to the Source tool during the modeling phase.

Multimedia Appendix 2 Recommendations on risk communication displayed in the e-learning training.

## Abbreviations

CHOICE: Choosing Treatment Together in Cancer at the End of Life
EORTC QLQ-C30: European Organisation for Research and Treatment of Cancer quality of life questionnaire C30
HRQoL: health-related quality of life
MoSCoW: Must Have, Should Have, Could Have, Won’t Have
SUS: System Usability Scale
TNM: tumor–node–metastasis

## References

[1] Ferlay J, Soerjomataram I, Dikshit R, Eser S, Mathers C, Rebelo M, Parkin DM, Forman D, Bray F (2015). Cancer incidence and mortality worldwide: sources, methods and major patterns in GLOBOCAN 2012. Int J Cancer.

[2] Badgwell B, Das P, Ajani J (2017). Treatment of localized gastric and gastroesophageal adenocarcinoma: the role of accurate staging and preoperative therapy. J Hematol Oncol.

[3] van Hagen P, Hulshof MC, van Lanschot JJ, Steyerberg EW, van Berge Henegouwen MI, Wijnhoven BP, Richel DJ, Nieuwenhuijzen GA, Hospers GA, Bonenkamp JJ, Cuesta MA, Blaisse RJ, Busch OR, ten Kate FJ, Creemers G, Punt CJ, Plukker JT, Verheul HM, Spillenaar Bilgen EJ, van Dekken H, van der Sangen MJ, Rozema T, Biermann K, Beukema JC, Piet AH, van Rij CM, Reinders JG, Tilanus HW, van der Gaast A, CROSS Group (2012). Preoperative chemoradiotherapy for esophageal or junctional cancer. N Engl J Med.

[4] Smyth EC, Verheij M, Allum W, Cunningham D, Cervantes A, Arnold D, ESMO Guidelines Committee (2016). Gastric cancer: ESMO Clinical Practice Guidelines for diagnosis, treatment and follow-up. Ann Oncol.

[5] Lordick F, Mariette C, Haustermans K, Obermannová R, Arnold D, ESMO Guidelines Committee (2016). Oesophageal cancer: ESMO Clinical Practice Guidelines for diagnosis, treatment and follow-up. Ann Oncol.

[6] van den Ende T, Ter Veer E, Machiels M, Mali RM, Nijenhuis FA, de Waal L, Laarman M, Gisbertz SS, Hulshof MC, van Oijen MG, van Laarhoven HW (2019). The efficacy and safety of (Neo) adjuvant therapy for gastric cancer: a network meta-analysis. Cancers (Basel).

[7] Ngai LL, ter Veer E, van den Boorn HG, van Herk EH, van Kleef JJ, van Oijen MG, van Laarhoven HW (2019). TOXview: a novel graphical presentation of cancer treatment toxicity profiles. Acta Oncol.

[8] ter Veer E, Mohammad NH, van Valkenhoef G, Ngai LL, Mali RM, Anderegg MC, van Oijen MG, van Laarhoven HW (2016). The efficacy and safety of first-line chemotherapy in advanced esophagogastric cancer: a network meta-analysis. J Natl Cancer Inst.

[9] ter Veer E, Mohammad NH, van Valkenhoef G, Ngai LL, Mali RM, van Oijen MG, van Laarhoven HW (2016). Second- and third-line systemic therapy in patients with advanced esophagogastric cancer: a systematic review of the literature. Cancer Metastasis Rev.

[10] van Kleef JJ, ter Veer E, van den Boorn HG, Schokker S, Ngai LL, Prins MJ, Mohammad NH, van de Poll-Franse LV, Zwinderman AH, van Oijen MG, Sprangers MA, van Laarhoven HW (2020). Quality of life during palliative systemic therapy for esophagogastric cancer: systematic review and meta-analysis. J Natl Cancer Inst.

[11] O'Connor AM, Légaré F, Stacey D (2003). Risk communication in practice: the contribution of decision aids. Br Med J.

[12] Stiggelbout AM, Pieterse AH, De Haes JC (2015). Shared decision making: Concepts, evidence, and practice. Patient Educ Couns.

[13] van den Ende T, ter Veer E, Mali RM, van Berge Henegouwen MI, Hulshof MC, van Oijen MG, van Laarhoven HW (2019). Prognostic and predictive factors for the curative treatment of esophageal and gastric cancer in randomized controlled trials: a systematic review and meta-analysis. Cancers (Basel).

[14] ter Veer E, van Kleef JJ, Schokker S, van der Woude SO, Laarman M, Mohammad NH, Sprangers MA, van Oijen MG, van Laarhoven HW (2018). Prognostic and predictive factors for overall survival in metastatic oesophagogastric cancer: a systematic review and meta-analysis. Eur J Cancer.

[15] Dugdale DC, Epstein R, Pantilat SZ (1999). Time and the patient-physician relationship. J Gen Intern Med.

[16] Stuij SM, Labrie NH, van Dulmen S, Kersten MJ, Christoph N, Hulsman RL, Smets E, INSTRUCT project group (2018). Developing a digital communication training tool on information-provision in oncology: uncovering learning needs and training preferences. BMC Med Educ.

[17] Politi MC, Han PK, Col NF (2007). Communicating the uncertainty of harms and benefits of medical interventions. Med Decis Making.

[18] Visser LN, Tollenaar MS, van Doornen LJ, de Haes HC, Smets EM (2019). Does silence speak louder than words? The impact of oncologists' emotion-oriented communication on analogue patients' information recall and emotional stress. Patient Educ Couns.

[19] Kessels RP (2003). Patients' memory for medical information. J R Soc Med.

[20] Clayton JM, Adler JL, O'Callaghan A, Martin P, Hynson J, Butow PN, Laidsaar-Powell RC, Arnold RM, Tulsky JA, Back AL (2012). Intensive communication skills teaching for specialist training in palliative medicine: development and evaluation of an experiential workshop. J Palliat Med.

[21] Politi MC, Légaré F (2010). Physicians' reactions to uncertainty in the context of shared decision making. Patient Educ Couns.

[22] McIntosh J (1974). Processes of communication, information seeking and control associated with cancer: a selective review of the literature. Soc Sci Med.

[23] Kodish E, Post SG (1995). Oncology and hope. J Clin Oncol.

[24] Good M, Pfleiderer B, Bibeau G (1991). The practice of biomedicine and the discourse on hope. Anthropologies of Medicine.

[25] Daugherty CK, Hlubocky FJ (2008). What are terminally ill cancer patients told about their expected deaths? A study of cancer physicians' self-reports of prognosis disclosure. J Clin Oncol.

[26] Douma KF, Koning CC, de Haes HC, Zandbelt LC, Stalpers LJ, Smets EM (2012). Do radiation oncologists tailor information to patients needs? And, if so, does it affect patients?. Acta Oncol.

[27] Oerlemans S, Husson O, Mols F, Poortmans P, Roerdink H, Daniels LA, Creutzberg CL, van de Poll-Franse LV (2012). Perceived information provision and satisfaction among lymphoma and multiple myeloma survivors - results from a Dutch population-based study. Ann Hematol.

[28] Hack TF, Degner LF, Parker PA (2005). The communication goals and needs of cancer patients: a review. Psychooncology.

[29] Puts MT, Papoutsis A, Springall E, Tourangeau AE (2012). A systematic review of unmet needs of newly diagnosed older cancer patients undergoing active cancer treatment. Support Care Cancer.

[30] Parameswaran R, McNair A, Avery KN, Berrisford RG, Wajed SA, Sprangers MA, Blazeby JM (2008). The role of health-related quality of life outcomes in clinical decision making in surgery for esophageal cancer: a systematic review. Ann Surg Oncol.

[31] Koedoot CG, Oort FJ, de Haan RJ, Bakker PJ, de Graeff A, de Haes JC (2004). The content and amount of information given by medical oncologists when telling patients with advanced cancer what their treatment options are. palliative chemotherapy and watchful-waiting. Eur J Cancer.

[32] Henselmans I, Jacobs M, van Berge Henegouwen MI, de Haes HC, Sprangers MA, Smets EM (2012). Postoperative information needs and communication barriers of esophageal cancer patients. Patient Educ Couns.

[33] Henselmans I, Smets EM, Han PK, de Haes HC, Laarhoven HW (2017). How long do I have? Observational study on communication about life expectancy with advanced cancer patients. Patient Educ Couns.

[34] Zandbelt LC, Smets EM, Oort FJ, Godfried MH, de Haes HC (2004). Satisfaction with the outpatient encounter: a comparison of patients' and physicians' views. J Gen Intern Med.

[35] Jacobs M (2015). Supporting patients in obtaining and oncologists in providing evidence-based health-related quality of life information prior to and after esophageal cancer surgery. PhD thesis.

[36] Hodgkinson K, Butow P, Hunt GE, Pendlebury S, Hobbs KM, Lo SK, Wain G (2007). The development and evaluation of a measure to assess cancer survivors' unmet supportive care needs: the CaSUN (Cancer Survivors' Unmet Needs measure). Psychooncology.

[37] Ravdin PM, Siminoff LA, Davis GJ, Mercer MB, Hewlett J, Gerson N, Parker HL (2001). Computer program to assist in making decisions about adjuvant therapy for women with early breast cancer. J Clin Oncol.

[38] Wishart GC, Azzato EM, Greenberg DC, Rashbass J, Kearins O, Lawrence G, Caldas C, Pharoah PD (2010). PREDICT: a new UK prognostic model that predicts survival following surgery for invasive breast cancer. Breast Cancer Res.

[39] van den Boorn HG, Engelhardt EG, van Kleef J, Sprangers MA, van Oijen MG, Abu-Hanna A, Zwinderman AH, Coupé VM, van Laarhoven HW (2018). Prediction models for patients with esophageal or gastric cancer: a systematic review and meta-analysis. PLoS One.

[40] Farmer G, Pearson G, Skylark W, Freeman A, Spiegelhalter D (2020). Making prognostic algorithms useful in shared decision-making: patients and clinicians requirements for the Predict: Breast Cancer interface. medRxiv.

[41] Butler L, Degner L, Baile W, Landry M, SCRN Communication Team (2005). Developing communication competency in the context of cancer: a critical interpretive analysis of provider training programs. Psychooncology.

[42] Bos-van den Hoek DW, Visser LN, Brown RF, Smets EM, Henselmans I (2019). Communication skills training for healthcare professionals in oncology over the past decade: a systematic review of reviews. Curr Opin Support Palliat Care.

[43] Kotter JP, Schlesinger LA (1979). Choosing strategies for change. Harv Bus Rev.

[44] Craig P, Dieppe P, Macintyre S, Michie S, Nazareth I, Petticrew M, Medical Research Council Guidance (2008). Developing and evaluating complex interventions: the new Medical Research Council guidance. Br Med J.

[45] van den Boorn HG, Abu-Hanna A, Mohammad NH, Hulshof MC, Gisbertz SS, Klarenbeek BR, Slingerland M, Beerepoot LV, Rozema T, Sprangers MA, Verhoeven RH, van Oijen MG, Zwinderman KH, van Laarhoven HW (2021). SOURCE: prediction models for overall survival in patients with metastatic and potentially curable esophageal and gastric cancer. J Natl Compr Canc Netw.

[46] van den Boorn HG, Abu-Hanna A, Ter Veer E, van Kleef JJ, Lordick F, Stahl M, Ajani JA, Guimbaud R, Park SH, Dutton SJ, Bang Y, Boku N, Mohammad NH, Sprangers MA, Verhoeven RH, Zwinderman AH, van Oijen MG, van Laarhoven HW (2019). SOURCE: A registry-based prediction model for overall survival in patients with metastatic oesophageal or gastric cancer. Cancers (Basel).

[47] Sivendran S, Latif A, McBride RB, Stensland KD, Wisnivesky J, Haines L, Oh WK, Galsky MD (2014). Adverse event reporting in cancer clinical trial publications. J Clin Oncol.

[48] van de Water LF, van Kleef JJ, Dijksterhuis WP, Henselmans I, van den Boorn HG, Morel NM, Schut KF, Daams JG, Smets EM, van Laarhoven HW (2020). Communicating treatment risks and benefits to cancer patients: a systematic review of communication methods. Qual Life Res.

[49] Nielsen J (1994). Enhancing the explanatory power of usability heuristics. Proceedings of the SIGCHI Conference on Human Factors in Computing Systems.

[50] Sommerville I (2016). Software Engineering 10th Edition (International Computer Science).

[51] Hakone A, Harrison L, Ottley A, Winters N, Gutheil C, Han P, Chang R (2017). PROACT: Iterative design of a patient-centered visualization for effective prostate cancer health risk communication. IEEE Trans Vis Comput Graph.

[52] Brooke J (1996). SUS: a quick and dirty usability scale. Usability Evaluation In Industry.

[53] Snijders T, Bosker R (1994). Case Method Fast Track: A Rad Approach.

[54] Villanueva R, Chen Z, Wickham H (2016). ggplot2 : Elegant Graphics for Data Analysis.

[55] Chang W, Cheng J, Allaire J, Xie Y, McPherson J (2017). Shiny: web application framework for R. R Package Version 1.6.0.

[56] Onwuegbuzie A, Dickinson W, Leech N, Zoran A (2009). A qualitative framework for collecting and analyzing data in focus group research. Int J Qual Methods.

[57] (2012). Think-aloud demonstration video. Gabor Aranyi PvSaPBoTU, UK (techslingtv).

[58] Kirschner P, van Merriënboer JJ Ten steps to complex learning : a new approach to instruction and instructional design. Citeseer.

[59] Van Merrienboer JJ, Reiser R, Dempsey JV (2007). Alternate models of instructional design: holistic design approaches and complex learning. Trends and Issues in Instructional Design and Technology (2nd Ed.).

[60] Van Merriënboer JJ, Kirschner PA (2001). Three worlds of instructional design: state of the art and future directions. Instr Sci.

[61] Henselmans I, Smets EM, de Haes JC, Dijkgraaf MG, de Vos FY, van Laarhoven HW (2018). A randomized controlled trial of a skills training for oncologists and a communication aid for patients to stimulate shared decision making about palliative systemic treatment (CHOICE): study protocol. BMC Cancer.

[62] Gilligan T, Coyle N, Frankel RM, Berry DL, Bohlke K, Epstein RM, Finlay E, Jackson VA, Lathan CS, Loprinzi CL, Nguyen LH, Seigel C, Baile WF (2017). Patient-clinician communication: American Society of Clinical Oncology consensus guideline. J Clin Oncol.

[63] Kiely BE, Soon YY, Tattersall MH, Stockler MR (2011). How long have I got? Estimating typical, best-case, and worst-case scenarios for patients starting first-line chemotherapy for metastatic breast cancer: a systematic review of recent randomized trials. J Clin Oncol.

[64] Kiely BE, Stockler MR, Tattersall MH (2011). Thinking and talking about life expectancy in incurable cancer. Semin Oncol.

[65] Zipkin DA, Umscheid CA, Keating NL, Allen E, Aung K, Beyth R, Kaatz S, Mann DM, Sussman JB, Korenstein D, Schardt C, Nagi A, Sloane R, Feldstein DA (2014). Evidence-based risk communication: a systematic review. Ann Intern Med.

[66] Sella T, Botser D, Navon R, Biran H, Tenenbaum S, Urban D, Onn A, Bar J (2015). Preferences for disclosure of disease related information among thoracic cancer patients. Lung Cancer.

[67] Hoffmann TC, Del Mar C (2015). Patients' expectations of the benefits and harms of treatments, screening, and tests: a systematic review. JAMA Intern Med.

[68] Henselmans I, van Laarhoven HW, de Haes HC, Tokat M, Engelhardt EG, van Maarschalkerweerd PE, Kunneman M, Ottevanger PB, Dohmen SE, Creemers G, Sommeijer DW, de Vos FY, Smets EM (2019). Training for medical oncologists on shared decision-making about palliative chemotherapy: a randomized controlled trial. Oncologist.

[69] Colevas A, Setser A (2004). The NCI Common Terminology Criteria for Adverse Events (CTCAE) v 3.0 is the new standard for oncology clinical trials. J Clin Oncol.

[70] Scott N, Fayers P, Aaronson N, Bottomley A, de Graef A, Groenvold M, Gundy C, Koller M, Petersen MA, Sprangers MA (2008). EORTC QLQ-C30 reference values manual. EORTC Quality of Life Group.

[71] Stiefel F, Barth J, Bensing J, Fallowfield L, Jost L, Razavi D, Kiss A (2010). Communication skills training in oncology: a position paper based on a consensus meeting among European experts in 2009. Ann Oncol.

[72] De Vries AM, de Roten Y, Meystre C, Passchier J, Despland J, Stiefel F (2014). Clinician characteristics, communication, and patient outcome in oncology: a systematic review. Psychooncology.

[73] Grossman R, Salas E (2011). The transfer of training: what really matters. Int J Train Dev.

[74] Cepeda NJ, Coburn N, Rohrer D, Wixted JT, Mozer MC, Pashler H (2009). Optimizing distributed practice: theoretical analysis and practical implications. Exp Psychol.

[75] Dempster F (1989). Spacing effects and their implications for theory and practice. Educ Psychol Rev.

[76] Dempster F, Perkins P (1993). Revitalizing classroom assessment: using tests to promote learning. J Instr Psychol.

[77] Donovan J, Radosevich D (1999). A meta-analytic review of the distribution of practice effect: now you see it, now you don't. J Appl Psychol.

[78] Moss V (1995). The efficacy of massed versus distributed practice as a function of desired learning outcomes and grade level of the student. All Graduate Theses and Dissertations - Utah State University.

[79] Janiszewski C, Noel H, Sawyer A (2003). A meta-analysis of the spacing effect in verbal learning: implications for research on advertising repetition and consumer memory. J Consum Res.

[80] van Kleef JJ, van den Boorn HG, Verhoeven R, Vanschoenbeek K, Abu-Hanna A, Zwinderman A, Sprangers MA, van Oijen MG, De Schutter H, van Laarhoven HW (2020). External validation of the Dutch Source Survival Prediction Model in Belgian metastatic oesophageal and gastric cancer patients. Cancers (Basel).

[81] Hillen MA, Gutheil CM, Smets EM, Hansen M, Kungel TM, Strout TD, Han PK (2017). The evolution of uncertainty in second opinions about prostate cancer treatment. Health Expect.

[82] van den Boorn HG, Stroes CI, Zwinderman AH, Eshuis WJ, Hulshof MC, van Etten-Jamaludin FS, Sprangers MA, van Laarhoven HW (2020). Health-related quality of life in curatively-treated patients with esophageal or gastric cancer: a systematic review and meta-analysis. Crit Rev Oncol Hematol.

[83] van den Ende T, Nijenhuis FA, van den Boorn HG, Ter Veer E, Hulshof MC, Gisbertz SS, van Oijen MG, van Laarhoven HW (2019). COMplot, a graphical presentation of complication profiles and adverse effects for the curative treatment of gastric cancer: a systematic review and meta-analysis. Front Oncol.

[84] (2019). Source Tool.

