# Supplementary material for: Informing Patients With Esophagogastric Cancer About Treatment Outcomes by Using a Web-Based Tool and Training: Development and Evaluation Study
Source: J Med Internet Res. 2021 Aug 27;23(8):e27824. doi: 10.2196/27824 (PMC8433928; doi:10.2196/27824)
Supplement: Multimedia Appendix 1 [file jmir_v23i8e27824_app1.docx]

| **Patients** |  |  |
| --- | --- | --- |
| **Comment** | **Support** | **Result** |
| Pictograph legend should be on top | 3 out of 6 | Pictograph legend is moved to top of graph. |
| Title is unclear when showing prediction for a certain month/year | 3 out of 6 | The title was changed to display e.g. 6 months instead of specific date. |
| Pictograph is clearer than line graph | 6 out of 6 | The pictograph is shown by default, the linegraph is optional. |
| The distance between bars in bargraph is too big | 4 out of 6 | The distance between bars is made smaller |
| Linegraph ic clearer than bargraph to display HRQoL | 4 out of 6 | HRQoL predictions are now only shown in a linegraph. |
| Unclear what mild/severe toxicity means | 4 out of 6 | In the training focus is given to explain this clearly to patients. |
| Don't use abbreviations of chemotherapy names | 1 out of 6 | Due to low support among patients, complex chemotherapy names and use of abbreviations in clinical practice, the abbreviations remain in use. |
|  |  |  |
| The text must be larger and clearer | 3 out of 6 | The font and font size was changed. |
| The meaning of the pictograph is unclear | 2 out of 6 | In the training focus is given to explain the pictographs clearly to patients. |
| Scenario function (best-case, typical outcome and worst-case) is difficult | 3 out of 6 | In the training focus is given to explain this clearly to patients. |
| Scenario function is helpful/important and relevant | 4 out of 6 | The scenario function remains in the tool and the training for physicians focuses on explaining the functionality. |
| Bars in the toxicity graph displaying mild and severe toxicity are interpreted as both stacked and non-stacked | 5 out of 6 | The bars displaying mild and severe toxicity are placed next to each other with over 90% overlap to clarify that the bars are not stacked. |
| The difference between mild and severe toxicity is too small | 3 out of 6 | The severe toxicity bars are made darker to increase contrast with mild toxicity. |
| The main application color is green which is not preferred | 3 out of 6 | The main application color is changed to a more neutral blue color. |
|  |  |  |
| **Physicians** |  |  |
| **Comment** | **Support** | **Result** |
| The input of the models requires a specific diagnosis date which ... | 3 out of 3 | The date input is removed from the tool and predictions are not shown for a specific date but rather in the form '6 months' |
| The label 'Esophagus NOS' is unclear | 1 out of 3 | The label is changed to 'Other' |
| The label 'lymph node metastasis' is unclear | 1 out of 3 | The label is changed to 'local lymph node metastasis' |
| Choosing the exact tumor location can be difficult in practice | 1 out of 3 | The descriptions of the tumor location have been changed and a visualisation of the stomach and esophagus have been added for extra clarity |
| There should be more input values on a single page | 1 out of 3 | The number of inputs on a page has increased to fill an entire screen |
| Show the survival in text next to the pictograph | 1 out of 3 | The survival has been added to the legend |
| Cannot find the function for most probable survival | 3 out of 3 | The function is now displayed in text instead of an icon and is clearly stated under diagram options |
| Missing median time when hovering over graph | 2 out of 3 | When hovering over de graph a dotted line is shown to indicate the time on the x-axis |
| Bars in the side effects graph displaying mild and severe side effects are interpreted as both stacked and non-stacked | 1 out of 3 | The bars displaying mild and severe side effects are placed next to each other with over 90% overlap to clarify that the bars are not stacked |
| Set side effects in order of decreasing occurence | 2 out of 3 | This suggestion was applied to the tool. However, it was found that it was confusing that a side effects would shift in the bar-graph with the addition of another treatment. All side effects now have a set order. |
| Pop up when no chemo is selected can be annoying | 2 out of 3 | The pop-up is removed. |
| The absence of a save button after filling in the models input values is confusing | 2 out of 4  (pilot study) | A save button is added. |
| The side effects graph is difficult to understand without extra explanation | 1 out of 4 (pilot study) | A clarifying picture is added as a legend to indicate which bars describe mild side effects and which are severe side effects. |
| The x-axis of the scenario graph is hard to read | 1 out of 4 (pilot study) | A hover function is added with a vertical line to the x-axis, in the future x-axis values are also displayed in the hover window. |
| Display the x-axis of the survival line graph in half year increments instead of two months increments | 1 out of 4 (pilot study) | When the x-axis range is set to 1 year, the x-axis is displayed in increments of one month. When the range extends 1 year, increments of half a year are displayed. |
|  |  |  |
| **Experts** |  |  |
| **Comment** |  | **Result** |
| The direct appearance of the survival graph after filling in the input values can be confronting for patients |  | After inputting the patient data, an empty screen is shown after which the physician can select an outcome to display. |
| Display the number of coloured icons in the pictograph in numbers too, above/on the side of the graph |  | The number of coloured icons is displayed in the legend above the graph. |
| Display the icons as overlapping when this is meant |  | When two treatments are compared, coloring an icon in two colors was found to be confusing, so the two treatments remain in a single pictograph. However, for three or more treatments, each treatment is displayed in a single pictograph. |
| Display the scale on the y-axis of the survival line graph as frequencies (100/100) instead of percentages (100%) |  | The suggested change was judged to make reading the graph confusing for high graph literacy people (i.e. physicians). |
| Accompanying verbal explanation of the scenario’s can be difficult for the physician to come up with him-/herself |  | An information button will be added to the scenario’s graph. |
| The reference score in the quality of life graph is not displayed in the legend |  | A label is added to the side of the graph |
| The side effects graph is hard to read: it is unclear whether the bars are stacked or not. |  | The display is changed from only side-to-side bars to overlapping bars, for the severe and mild side effects to clarify that the bars are not stacked. |
| The automatically generated texts for the summary function are difficult to understand for low literacy patients due to terminology |  | Difficult terminology is replaced by easy to understand alternatives where possible. Abstract concepts (i.e. quality of life) are explained in the summary text. |
| The summary function should have display options of its own |  | Summary display options are added, but turn out to make the summary page more unclear by an overload of options. Also, the website speed is decreased a lot by this added functionality. The options are therefore removed and the preferences as set in the main graph on the outcome tab are also displayed in the summary. |
