# Supplementary material for: Informing Patients With Esophagogastric Cancer About Treatment Outcomes by Using a Web-Based Tool and Training: Development and Evaluation Study
Source: J Med Internet Res. 2021 Aug 27;23(8):e27824. doi: 10.2196/27824 (PMC8433928; doi:10.2196/27824)
Supplement: Multimedia Appendix 2 [file jmir_v23i8e27824_app2.docx]

**Recommendations on risk communication displayed in the e-learning**

1. Do not use only general verbal descriptions (e.g. ‘often’, ‘some people’), but add numbers (e.g. 50% or 50/100) to your description of the risk or benefit. Solely verbal descriptions might easily be misunderstood or misjudged by patients.
2. Accompany your information with visual representations of the information to facilitate the understanding, for instance by using the Source tool.
3. Do not use relative risk reductions. Research shows that these are often more often misunderstood than absolute risk reductions and that relative risk reductions can influence treatment choice.
4. Preferably use frequencies and/or percentages to inform patients on risks and benefits of treatment. Be sure to use clear descriptions that cannot be easily misunderstood.
5. Add a reference class to your frequency/percentage (time frame, place, total number of people, etc.). To achieve this ask yourself the following questions:
   1. Whom does this number apply to?
   2. Which period of time or moment in time does this number apply to?
6. Use the three scenarios as introduced by Kiely et al. to inform about survival. These scenario’s stress the range of outcomes allowing patients to prepare for the worst and hope for the best.
